# Nanoscopy at low light intensities shows its potential

**DOI:** 10.7554/eLife.00475

**Published:** 2012-12-31

**Authors:** Travis J Gould, Joerg Bewersdorf

**Affiliations:** 1 **Travis J Gould** is in the Department of Cell Biology and the Kavli Institute for Neuroscience, Yale University School of Medicine, New Haven, United Statestravis.gould@yale.edu; 2 **Joerg Bewersdorf** is in the Departments of Cell Biology and Biomedical Engineering, and the Kavli Institute for Neuroscience, Yale University School of Medicine, New Haven, United Statesjoerg.bewersdorf@yale.edu

**Keywords:** confocal microscopy, fluorescent probes, GFP, nanoscopy, superresolution, live-cell imaging, None

## Abstract

A new form of green fluorescent protein allows super-resolution imaging to be performed faster on living cells with low radiation doses.

**Related research article** Grotjohann T, Testa I, Reuss M, Brakemann T, Eggeling C, Hell SW, Jakobs S. 2012. rsEGFP2 enables fast RESOLFT nanoscopy of living cells. *eLife* **1**:e00248. doi: 10.7554/eLife.00248

**Image** Super-resolution image of a live mammalian cell

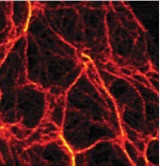

The availability of fluorescence microscopes that can image sub-cellular structures labelled by fluorescent markers in living cells has provided fresh insights into a variety of cellular processes and functions. For most of the 20th century the resolution of such lens-based microscopes was limited by diffraction of the light used to form the image. This diffraction limit is about half the wavelength of the light, which means that an optical microscope cannot achieve a resolution any better than about 250 nm. In 1994, however, Stefan Hell and Jan Wichmann predicted that the diffraction limit could be beaten by a new form of fluorescence microscopy called stimulated emission depletion (STED) microscopy (Hell and Wichmann, 1994). This approach and related techniques are now routinely used to image fixed and living cells with a resolution of better than 100 nm (Gould et al., 2012). However, the high light intensities needed for these forms of ‘nanoscopy’, combined with relatively slow imaging speeds, can be problematic when imaging living samples. Now, in *eLife*, Hell and co-workers report that a new fluorescent marker called rsEGFP2 allows them to image living cells at low light intensities some 25–250 times faster than was possible previously (Grotjohann et al., 2012).

In fluorescence microscopy, fluorescent markers (also known as fluorophores) are excited by a bright light source, often a laser. After a short time the excited fluorophore returns to its electronic ground state by releasing a fluorescence photon, and an image of the sample can be built up by detecting these photons. Whereas the resolution of a conventional microscope is determined by the optics of the instrument, the resolution achievable with a fluorescence nanoscope depends on both the optics and the properties of the fluorophore. The diffraction limit is overcome by switching the fluorophores in the sample on and off (Hell, 2009). In STED microscopy, for example, a second laser is used to transfer the fluorophore from the excited state to the ground state without fluorescing. By focusing the second laser (the depletion laser) in a ring shape, and then overlaying this pattern on the first laser, it is possible to effectively switch off all of the fluorophores, apart from those very close to the centre of the ring (where the intensity of the depletion laser is negligible; Figure 1). This targeted switching approach thereby creates a sub-diffraction sized region from which fluorescence can be emitted. A ‘super-resolution’ image is built up by scanning the two laser foci across the sample and recording the fluorescence signal.

**Figure 1.**
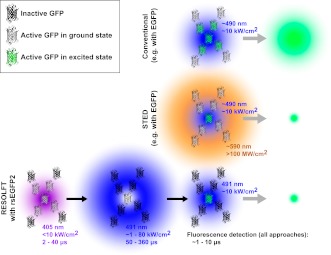
Comparison of three forms of fluorescence microscopy. In conventional fluorescence microscopy (top), a single laser (shown here in blue) is used to excite green fluorescent protein (GFP) or some other fluorophore, and the resulting fluorescence is detected. The resolution is determined by the size of region in which the GFP is excited (shown in green) and corresponds to the diffraction-limited laser focus size. In stimulated emission depletion (STED) nanoscopy (middle), a second ring-shaped laser focus (shown here in orange) is used to transfer excited GFP molecules at the periphery of the excitation focus back to the ground state without fluorescing: the size of the region in which the GFP can still fluoresce is smaller than in conventional fluorescence microscopy and is no longer limited by the laser focus size. In RESOLFT nanoscopy with rsEGFP2 (bottom), inactive fluorophores (shown as black molecular structures) are first activated by a violet laser focus. A second blue ring-shaped laser focus then continuously excites activated fluorophores until they have switched back to the inactive state. After these two steps, a conventional blue laser focus is used to illuminate the remaining active molecules and the resulting fluorescence is detected. Similar to STED nanoscopy, the size of the region in which rsEGFP2 can still fluoresce is not limited by diffraction. Typical wavelengths, laser intensities, and illumination times are shown for each laser. The period during which fluorescence is detected by all three methods is comparable (∼1 to 10 μs). Note that GFPs are not shown to scale. EGFP is enhanced GFP.

The resolution of STED microscopes is given by the equation Δr ≈ λ/(2NA(1+*I*_max_/*I*_sat_)^1/2^), where λ is the wavelength of the depletion laser, NA is the numerical aperture of the objective lens, *I*_max_ is the peak intensity of the depletion laser, and *I*_sat_ is the saturation intensity of the fluorophore (that is, the intensity at which 50% of the fluorescence is depleted; Hell, 2009). The size of the fluorescent region can, in principle, be made arbitrarily small by increasing the peak intensity of the depletion laser *I*_max_ or by using a fluorophore with a low saturation intensity *I*_sat_, which can be achieved by using a fluorophore with a long excited state lifetime *T*_fluor_ (because *I*_sat_ is inversely proportional to *T*_fluor_). Due to the short lifetimes of most fluorophores, which are on the order of nanoseconds, the saturation intensity *I*_sat_ is typically ∼10^4^ kW/cm^2^ (Hell, 2009). This means that the peak intensity of the depletion laser *I*_max_ needs to be higher than ∼10^5^ kW/cm^2^ to push the resolution significantly below the diffraction limit. In addition to limiting laser choice, such high intensities raise concerns that living specimens could suffer long-term photodamage.

Targeted switching can, in principle, be used with any reversibly switchable optical transition, not just stimulated emission, as described by the RESOLFT principle (Hell et al., 2003). By choosing a transition with a lifetime that is much longer than *T*_fluor_, it is possible to push the resolution well below the diffraction limit with laser intensities much lower than those used in STED microscopy. One variant of RESOLFT nanoscopy has recently come to fruition in biological imaging experiments using fluorescent proteins that can be reversibly switched between two states (Brakemann et al., 2011; Grotjohann et al., 2011; Testa et al., 2012).

These approaches require three illumination steps: one for activation, one for targeted deactivation and one for readout of the remaining activated probe molecules. For example RESOLFT nanoscopy using rsEGFP (reversibly switching enhanced green fluorescent protein) works as follows: first, a diffraction-limited violet laser focus is used to activate the fluorophores (i.e., switch them to a state that is capable of fluorescence). Second, a ring-shaped blue laser focus repeatedly excites the activated molecules to fluoresce, although this fluorescence is not recorded, and eventually deactivates them (i.e., switches them to a fluorescence incapable state). Laser light of the same colour, but conventionally focused, is then used to excite the remaining activated fluorophores at the centre of the ring and the fluorescence from this sub-diffraction area is detected. This three-step process is repeated for every pixel of the super-resolution image. Due to the long lifetime of the activated state, low intensities of the ring-shaped focus of *I*_max_ ∼1 to 10 kW/cm^2^ have been sufficient to achieve sub-100 nm resolution. However, the long pixel dwell times required by the slow switching kinetics of the fluorophore, rsEGFP, have led to image acquisition times of up to about an hour in the past (Grotjohann et al., 2011).

Now Hell and co-workers—including Tim Grotjohann and Ilaria Testa of the Max Planck Institute for Biophysical Chemistry as joint first authors, and Stefan Jakobs as joint corresponding author—report that they have modified this protein to make a new fluorophore called rsEGFP2 with a shorter switching time and improved resistance to switching fatigue (Grotjohann et al., 2012). This has allowed them to image various structures inside living cells 25–250 times faster than previously reported. Importantly, the relatively low laser intensities (∼1 to 80 kW/cm^2^ in a laser scanning configuration in which every point of the sample is illuminated for only a fraction of the frame acquisition time) needed to switch off the fluorescence mean that this approach exposes samples to the lowest radiation doses of all the nanoscopy techniques demonstrated to date.

Compared to STED nanoscopy, the current approach to RESOLFT nanoscopy requires additional activation and deactivation steps before the actual fluorescence can be recorded (see Figure 1). These steps inherently add overhead to the imaging process, and this slows down data acquisition. However, the advent of new fluorescent proteins such as rsEGFP2 extends RESOLFT nanoscopy with photoswitchable markers to faster live-cell imaging and holds great promise for future biological applications. The use of low light intensities results in reduced phototoxicity and is likely to allow imaging over extended time periods. Temporal resolution can be further improved using parallelization techniques and, if combined with faster switching rates, this should lead to video-rate RESOLFT nanoscopy over large fields of view.
